# Neutrophil Chemotaxis and NETosis in Murine Chronic Liver Injury via Cannabinoid Receptor 1/Gα_i/o_/ROS/p38 MAPK Signaling Pathway

**DOI:** 10.3390/cells9020373

**Published:** 2020-02-05

**Authors:** Xuan Zhou, Le Yang, Xiaoting Fan, Xinhao Zhao, Na Chang, Lin Yang, Liying Li

**Affiliations:** Department of Cell Biology, Municipal Laboratory for Liver Protection and Regulation of Regeneration, Capital Medical University, Beijing 100069, China; zhouxuanyee@126.com (X.Z.); yangle@ccmu.edu.cn (L.Y.); 18210564428@163.com (X.F.); xinhaozhao0010@163.com (X.Z.); changna@ccmu.edu.cn (N.C.); yang_lin@ccmu.edu.cn (L.Y.)

**Keywords:** liver injury, neutrophil extracellular trap, myeloperoxidase, carbon tetrachloride

## Abstract

Neutrophils play an essential role in the control of inflammatory diseases. However, whether cannabinoid receptors (CBs) play a role in neutrophil chemotaxis and NETosis in sterile liver inflammation remains unknown. The expression of marker genes on neutrophils was characterized by FACS, immunofluorescence, qRT-PCR, and Western blot. The amount of neutrophils was significantly elevated from 7 days and reached the peak at 2 weeks in carbon tetrachloride (CCl_4_)-treated mouse liver. The mRNA expression of neutrophil marker Ly6G had positive correlation with CB1 and CB2 expression in injured liver. In vitro CBs were abundantly expressed in isolated neutrophils and CB1 agonist ACEA promoted the chemotaxis and cytoskeletal remodeling, which can be suppressed by CB1 antagonist AM281. Moreover, ACEA induced NETosis, myeloperoxidase release from lysosome and ROS burst, indicating neutrophil activation, via Gα_i/o_. Conversely, CB2 agonist JWH133 had no effect on neutrophil function. ROS and p38 MAPK signaling pathways were involved in CB1-mediated neutrophil function, and ROS was upstream of p38 MAPK. CB1 blockade in vivo significantly attenuated neutrophil infiltration and liver inflammation in CCl_4_-treated mice. Taken together, CB1 mediates neutrophil chemotaxis and NETosis via Gα_i/o_/ROS/p38 MAPK signaling pathway in liver inflammation, which represents an effective therapeutic strategy for liver diseases.

## 1. Introduction

Neutrophils are the most abundant white blood cells and among the first cells recruited to an inflammatory site, thus mediating the early responses to tissue injury [1]. Neutrophil activation is characterized by neutrophil extracellular trap (NET) formation (NETosis) [2,3], granule enzymes myeloperoxidase (MPO) release from lysosome (azurophilic granules) [4] and ROS burst [5], further contributes to inflammation-associated damage in injured tissue. NETosis was first described in 2004 as highly decondensed chromatin structures, which was associated with citrullination of histone H3 [6,7]. NETosis has been found in response to various stimuli such as LPS, damage-associated molecular patterns (DAMPs), and PMA [6,8]. Recent studies have implicated that neutrophils play an essential role in the control of sterile inflammatory diseases, which are also characterized by a sustained influx of neutrophils and persistent NET release, and contribute to various injury processes [9,10]. For instance, in chronic obstructive pulmonary disease and cystic fibrosis, neutrophils and NETs contribute to chronic inflammatory and lung tissue damage [11,12]. Peptidylarginine deiminase (PAD) inhibition reduces NETosis and protects against lupus-related vasculature, kidney and skin injury in various lupus models [13]. In pyogenic arthritis, pyoderma gangrenosum and acne syndrome, an imbalance of NET formation and degradation are detected that enhances the half-life of these structures in vivo and promotes inflammation [14]. Especially, the important role of neutrophils has been identified in acute liver injury. In liver ischemia/reperfusion, interleukin-33, which is released from liver sinusoidal endothelial cells, promotes NETosis of infiltrating neutrophils and exacerbates inflammatory injury [15]. Disruption of the miR-223 gene exacerbates acetaminophen-induced hepatic neutrophil infiltration, oxidative stress, and injury, and enhances TLR9 ligand-mediated activation of pro-inflammatory mediators in neutrophils [16]. However, it remains unknown about the mediator and underlying molecular mechanism of regulating neutrophil recruitment and activation during chronic liver injury.

The endocannabinoid system (ECS) comprises cannabinoid receptors (CBs; CB1 and CB2), endocannabinoids and their synthesis and degradation enzymes [17]. CB1 is highly expressed in central nervous system and is also found in the periphery, including immune system and liver at a lower level, while CB2 is mainly expressed in immune cells [18]. ECS has been proven to be involved in the regulation of multiple physiological processes, such as appetite control, energy balance, pain perception, and immune response [19,20,21]. Notably CBs have been identified as pivotal regulators of acute and chronic liver injury, especially in inflammation-related liver injury [22]. For examples, a potential impact of CB1 on the inflammatory response associated with NASH has been suggested by experiments in obese rats, showing that CB1 antagonist rimonabant plays a hepatoprotective role in the treatment of obesity-associated liver diseases and related features of metabolic syndrome [23,24]. Moreover, CB1 and CB2 participate in the resveratrol-induced anti-NASH effect by maintaining the gut barrier integrity and inhibiting gut inflammation in high-fat diet-induced NASH rat models [25]. Our previous studies have also found that CB1 promotes the infiltration and activation of bone marrow (BM)-derived monocytes/macrophages in carbon tetrachloride (CCl_4_)-induced liver injury mouse model, which could be inhibited by the blockade of CB1 [26,27]. However, the knowledge of whether CBs are involved in neutrophil function during sterile liver inflammation remains limited. 

Here we investigate the effects of CBs on neutrophil chemotaxis and activation in isolated neutrophils and CCl_4_-induced murine models. Our findings suggest that CB1 but not CB2 mediates neutrophil chemotaxis and NETosis in vitro, which are dependent of ROS and MAPK signaling pathways. Furthermore, blockade of CB1 in vivo reduces the infiltration and activation of neutrophils and attenuates liver injury in CCl_4_-treated mice, which may represent an effective therapeutic strategy for liver diseases.

## 2. Materials and Methods

### 2.1. Materials 

RPMI Medium 1640 was from GIBCO/Invitrogen (Grand Island, NY, USA). PCR reagents were from Applied Biosystems. ACEA (special CB1 agonists), AM281 (CB1 antagonist), JWH133 (CB2 antagonist), SB203580 (p38 inhibitor) were from TOCRIS/R&D (Minneapolis, MN, USA). NAC and PTX were from Sigma-Aldrich (St. Louis, MO, USA). Fibronectin was from Calbiochem (Germany). YM254890 was from Adipogen Corp. (San Diego, CA, USA). SYTOX Green Nucleic Acid Stain was from Molecular Probes, Inc. (Eugene, OR, USA).

### 2.2. Mouse Models of Liver Fibrosis 

A CCl_4_ (1 µL/g BW)/OO mixture (1:9 v/v) was injected into abdominal cavity of mice twice per week. Mice were sacrificed at 1, 2, and 3 days and 1, 2, and 4 weeks. The liver tissues were harvested. The intraperitoneal injection of AM281 (2.5 mg/kg BW) or DNAase I (11284932001, 50 μg/mouse, Roche, Swiss) was performed at 4 or 24 h before CCl_4_ administration. All animal work was conformed to the Ethics Committee of Capital Medical University and in accordance with the approved guidelines (approval number: AEEI-2014-131).

### 2.3. BM Transplantation

ICR male mice aged 6 weeks received lethal irradiation (8 Grays) and immediately received transplantation by a tail vein injection of 1.5 × 10^7^ whole BM cells obtained from 3-week-old EGFP transgenic mice. Four weeks later, mice of BM-rebuild were subjected to CCl_4_-induced liver injury. After another 2 weeks, mice were sacrificed and liver tissues were harvested. 

### 2.4. FACS

Non-parenchymal cells of mouse liver were isolated as described previously [28]. APC-Ly6G (BD Biosciences, Franklin Lakes, NJ, USA) and its isotype-matched negative control were added to the non-parenchymal cell suspension, respectively. After 15 min incubation in the dark, the cells were washed with PBS and subjected to FACS, which was performed on a FACSAria and analyzed with FACS Diva 4.1 (BD, Biosciences).

### 2.5. Isolation of Mouse BM Neutrophils

ICR mice aged 6 weeks were sacrificed by cervical dislocation at the time of neutrophils harvest. Tibias and femurs were removed and stripped of their muscles. The BM was flushed using PBS, and cell aggregates were disrupted via filtration through 70-μm cell strainer (BD Bioscience) and washed with PBS. Cell suspension was layered in a ratio of 1 to 3 on top of Histopaque 1077 (Sigma Aldrich), after centrifugation, precipitate was resuspended the with PBS. The cell suspension was layered in a ratio of 1 to 2 on top of Histopaque 1119 (Sigma Aldrich), after centrifugation, neutrophils were recovered on the top of Histopaque 1119. Neutrophils were washed with PBS and then resuspended in RPMI Medium 1640. The purity of neutrophils was determined by immunofluorescence staining for Ly6G (almost 100% cells were positive for Ly6G). Neutrophil viability was analyzed using Cell Counting Kit-8 (CCK-8) (Dojindo, Kumamoto, Japan) according to the manufacturer’s procedure.

### 2.6. Neutrophils Chemotaxis Assay

Isolated bone marrow neutrophils were incubated with Calcein-AM (Life Technologies, CA, USA) to label cells and treated with AM281 (10 μM), NAC (5 mM), PTX (5 ng/mL), YM254890 (10 μM) or SB203580 (10 μM) for 20 min, then seeded to the upper chambers of a 3 μm-transwell (Corning). Then cells were allowed to migrate for another 2 h in the presence of ACEA (1 μM) or JWH133 (1 μM) in the lower chambers. The chambers were incubated at 37 °C in 5% CO_2_. Subsequently, chemotaxis of neutrophils was determined by the fluorescence value of Calcein-AM in the lower chambers, using a fluorescent plate reader EnVision 2104-0010 (Perkinelmer, MA, USA).

### 2.7. Western Blot Analysis 

Proteins were extracted from cells (50 μg) or liver tissue (100 μg) using RIPA Lysis Buffer (R0010, Solarbio, China) added with Complete Protease Inhibitor Cocktail Tablets (04693116001, Roche, Swiss). The components of RIPA Lysis Buffer were as follows: 50 mM Tris (pH 7.4), 150 mM NaCl, 1%TritonX-100, 1% sodium deoxycholate, 0.1% SDS, 2 mM sodium pyrophosphate, 25 mM b-glycerophosphate, 1 mM EDTA, 1mM Na_3_VO_4_, 0.5 mg/mL leupeptin. Then the extract was separated by SDS-PAGE and subjected to Western blot analysis. Membranes were incubated overnight using the following antibodies: rabbit anti-citrullinated-histone H3 (CitH3) polyclonal antibody (ab5103, 1:200, Abcam, Cambridge, United Kingdom), rabbit anti-ERK1/2 monoclonal antibody (4695) and rabbit anti-phorspho-ERK1/2 monoclonal antibody (4376, 1:1000, Cell Signaling, Beverly, MA, USA); rabbit anti-p38 polyclonal antibody (9212) and rabbit anti-phorspho-p38 polyclonal antibody (9211, 1:1000, Cell Signaling); rabbit anti-JNK polyclonal antibody (9252) and rabbit anti-phospho-JNK monoclonal antibody (4668, 1:1000, Cell Signaling); mouse anti-β-tubulin (HC101, 1:1000, TransGen Biotech, China) and anti-β-actin monoclonal antibodies (HC201, 1:1000, TransGen Biotech, China). IRDye 800CW Goat anti-Mouse IgG (H + L) Secondary Antibody (92632210), IRDye 800CW Goat anti-Rabbit IgG (H + L) Secondary Antibody (92632211, 1:10000, LI-COR, NE, USA) were used. The bands were displayed using ODYSSEY and quantified by Odyssey v3.0 software. β-tubulin or β-actin were as references.

### 2.8. Immunofluorescence Staining

Isolated BM neutrophils were plated to adhere in fibronectin-coated 96-well plates (Corning) or Nunc glass base dishes (Thermo Fisher Scientific, MA, USA) and pretreated with vehicle, AM281 (10 μM), NAC (5 mM), PTX (5 ng/mL), YM254890 (10 μM) or SB203580 (10 μM) for 20 min, respectively, and stimulated for 2 h with ACEA or JWH133. Neutrophils were fixed by 4% paraformaldehyde for 30 min, after blocked with 2% BSA (Roche, Switzerland), they were incubated with the specific primary antibodies for CitH3 (1:200), MPO (1:200) or Ly-6G (Clone 1A8, 551459, 1:100, BD pharmingen), CB1 (10006590, 1:200, Cayman Chemical, Ann Arbor, MI, USA), CB2 (1:200, 101550, Cayman Chemical) or Rabbit IgG Isotype Control (1:100, 10500C, Invitrogen, CA, USA). Cy3-conjugate affinipure goat-anti-rabbit IgG (111165003, 1:100) or Cy5-conjugate affinipure goat-anti-rat IgG (112175143, 1:100, Jackson Immunoresearch, PA, USA) were as secondary antibodies. For F-actin, neutrophils were fixed by 4% paraformaldehyde for 30 min, penetrated by 0.5% Triton X-100 for 15 min and after blocked with 2% BSA, FITC-conjugated phalloidin (A12379, 1:100, Molecular Probes, OR, USA) was incubated for 20 min. Nuclei were stained with DAPI and SYTOX Green. The sample was observed under confocal microscope (LSM510, Carl Zeiss MicroImaging GmbH, Germany). For high content analysis, the plates were imaged on Thermo Scientific CellInsight personal cell imaging platform (Thermo Fisher Scientific). Fluorescence intensity of each well was analyzed by Cellomics Cell Health Profiling BioApplication software.

### 2.9. RT-qPCR

Total RNA was extracted from liver frozen specimens with or without treatments using an RNeasy kit (Qiagen, Germany). RT-qPCR was performed in an ABI Prism 7300 sequence detecting system (Applied Biosystems, CA, USA). Primers were as follows: 18s rRNA: Sense, 5′-GTA ACC CGT TGA ACC CCA TT-3′; Antisense, 5′-CCA TCC AAT CGG TAG TAG CG-3′. Ly6G: Sense, 5′-AGA AGC AAA GTC AAG AGC AAT CTC T-3′; Antisense, 5′-TGA CAG CAT TAC CAG TGA TCT CAG T-3′. CB1: Sense, 5′-GGC GGT GGC CGA TCT C-3′; Antisense, 5′-CGG TAA CCC CAC CCA GTT T-3′. CB2: Sense, 5′-AGC GCC CTG GAG AAC ATG-3′; Antisense, AGC TGC TGA TGA ACA GGT ACG A-3′. MCP-1: Sense, 5′-TCT GGG CCT GCT GTT CAC A-3′; Antisense, 5′-GGA TCA TCT TGC TGG TGA ATG A-3′. IL-1β: Sense, 5′-GCA ACT GTT CCT GAA CTC AAC T-3′; Antisense, 5′-ATC TTT TGG GGT CCG TCA ACT-3′. IL-6: Sense, 5′-TAG TCC TTC CTA CCC CAA TTT CC-3′; Antisense, 5′-TTG GTC CTT AGC CAC TCC TTC-3′. CD86: Sense, 5′-TCC AAG TTT TTG GGC AAT GTC-3′; Antisense, 5′-CCT ATG AGT GTG CAC TGA GTT AAA CA-3′. 

### 2.10. ROS Production

2′,7′-Dichlorofluorescin diacetate (DCFHDA) (Sigma Aldrich) is a cell-permeable non-fluorescent probe, which is de-esterified intracellularly and turns to highly fluorescent 2′,7′-dichlorofluorescein upon oxidation. Isolated neutrophils were incubated with DCFHDA for 20 min and after seeded to 96-well plate, they were treated with different stimulators. The plate was then transferred onto a fluorescent plate reader, EnVision 2104-0010 (Perkinelmer, MA, USA), and detected the fluorescent value or Thermo Scientific CellInsight personal cell imaging platform to acquire ROS immunofluorescence images. 

### 2.11. Liver Damage Assessment

Serum ALT and AST levels were detected by BS-200 Chemistry Analyzer (MINDARY, China).

### 2.12. Histology Analysis

Liver tissues were fixed in 4% paraformaldehyde. Liver tissue sections (5 μm) were stained with H&E for assessment of liver inflammation and injury. The inflammatory response was quantified by calculating inflammatory area using Image J software. Fifteen randomly selected areas per sample were measured as the mean value of the expressed percentage of inflammatory area.

### 2.13. Statistical Analysis

The results are expressed as mean ± standard error of the mean (SEM). Statistical significance was assessed by Student’s t-test or one-way ANOVA for analysis of variance when appropriate. Correlation coefficients were calculated by Pearson test. *p* < 0.05 was considered to be significant. All results were verified in at least three independent experiments.

## 3. Results

### 3.1. Numerous Neutrophils Are Recruited and Activated in the Liver of CCl_4_-Treated Mice

**Figure 1:**
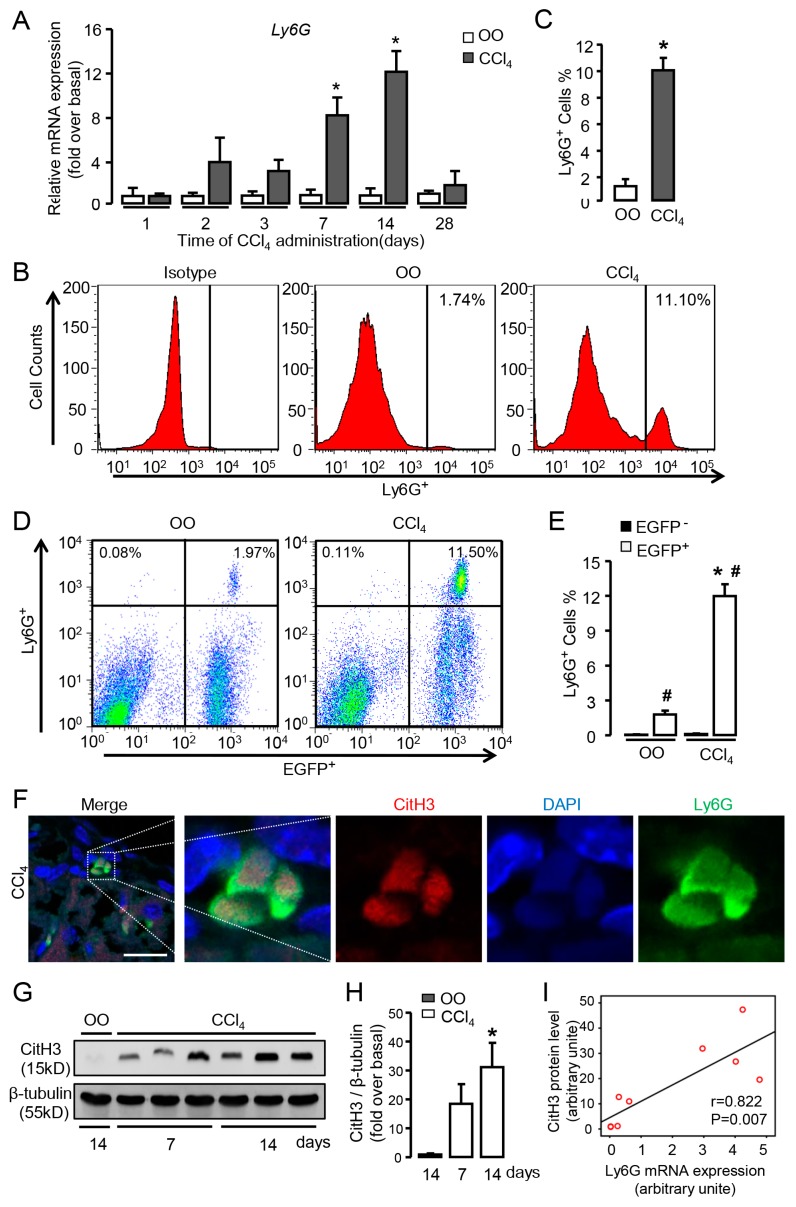
Numerous neutrophils are recruited and activated in the liver of carbon tetrachloride (CCl_4_)-treated mice. (**A**) The mRNA expression of neutrophil marker Ly6G was examined by qRT-PCR in the injured liver of CCl_4_ mice. (**B**,**C**) Representative FACS plots and quantification for total neutrophils (Ly6G^+^). (**D**,**E**) Representative FACS plots and quantification for neutrophils of BM origin (Ly6G^+^EGFP^+^) and non-BM origin (Ly6G^+^EGFP^𢈒^). (**F**) Immunofluorescent staining for CitH3 in the liver of CCl_4_-treated mice. Scale bars, 20 μm. (**G**,**H**) CitH3 expression in the injured liver was examined by Western blot. (**I**) The correlation between CitH3 protein levels and Ly6G mRNA expression in liver tissue. Data are presented as the mean ± SEM. N = 6 per group. * *p* < 0.05 vs. control. # *p* < 0.05 vs. EGFP^𢈒^ neutrophils with the same treatment.

To investigate the dynamic change of neutrophil signatures in sterile liver inflammation, we examined the mRNA expression of neutrophil marker Ly6G in the liver treated by CCl_4_ for different time points. Our results showed that Ly6G mRNA expression up-regulated from 7 days of CCl_4_ administration and reached the peak at 2 weeks, whereas the expression evidently decreased at 4 weeks compared with 2 weeks (Figure 1A), indicating that numerous neutrophils were recruited to injured liver during the early stage of chronic liver injury. Further, FACS analysis revealed that percentage of Ly6G^+^ neutrophils was much higher in CCl_4_-treated mice for 2 weeks compared with that in olive oil (OO)-treated mice (Figure 1B,C). 

To clarify the origin of neutrophils recruited to the injured liver, we performed a genetic EGFP-labeled BM cell transplantation to the mice that had been lethally irradiated. Then the chimeric mice received intraperitoneal injection of CCl_4_ for 2 weeks to induce liver injury. We isolated hepatic non-parenchymal cells from liver tissue and detected Ly6G^+^ cells by FACS. The percentage of Ly6G^+^EGFP^+^ neutrophils (BM origin, OO group: 1.81%; CCl_4_ group: 12.00%) was much higher than Ly6G^+^EGFP^𢈒^ neutrophils (non-BM origin, OO group: 0.07%; CCl_4_ group: 0.13%) in both OO and CCl_4_ groups (Figure 1D,E). Moreover, Ly6G^+^EGFP^+^ neutrophils were significantly increased after CCl_4_ administration compared with that in OO group (Figure 1D,E), indicating the recruited neutrophils in injured liver were mostly derived from BM. Then we performed immunofluorescent staining to examine CitH3 expression in the neutrophils of injured liver (Figure 1F). Further, increased hepatic level of citrullinated histone H3 (CitH3, specific marker of NETosis) was detected in CCl_4_-treated mice (Figure 1G,H), suggesting the activation of these infiltrating neutrophils in the injured liver. Correlation analysis showed a positive correlation between CitH3 protein levels and Ly6G mRNA expression in liver tissue (Figure 1I). Altogether these results demonstrate that large numbers of BM-derived neutrophils are recruited and activated in the early stage of chronic liver injury.

### 3.2. CB Expression Positively Correlates with Neutrophil Signatures in CCl_4_-Treated Mice, and CBs Are Abundantly Expressed in Isolated Neutrophils

**Figure 2:**
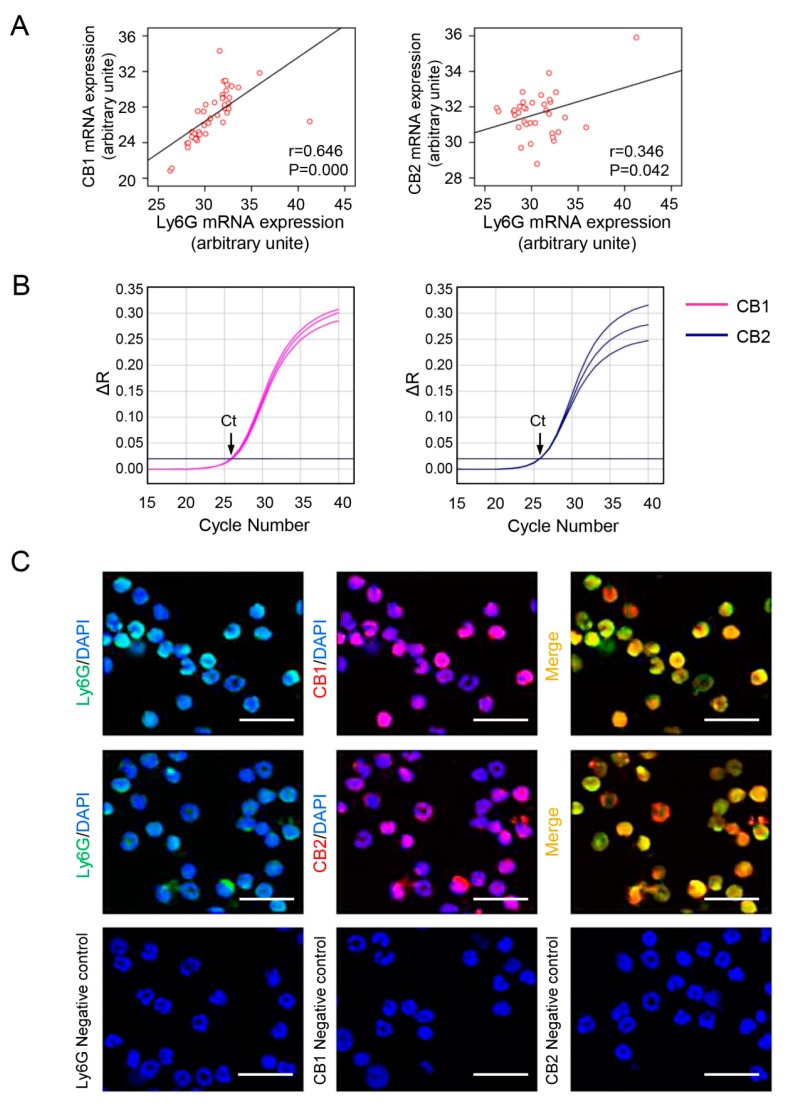
Cannabinoid receptor (CB) expression positively correlates with neutrophil signatures in CCl_4_-treated mice, and CBs are abundantly expressed in isolated neutrophils. (**A**) The correlation between Ly6G and CB1 or CB2 in liver tissue. (**B**) The amplification plots of CB1 and CB2 expression in neutrophils by RT-qPCR. (**C**) Representative images of immunofluorescent staining for Ly6G (green) and CB1 or CB2 (red) in neutrophils. The nuclei were stained with DAPI (blue). Scale bars, 20 μm. N = 6 per group.

Our previous study had showed that CB1 and CB2 expression were increased in CCl_4_-induced liver injury [27]. Here we undertook correlation analysis of mRNA expression levels between CB1 or CB2 and Ly6G. Each dot represented one liver sample from all mice (including OO and CCl_4_-treated groups). Correlation coefficients were calculated using relative mRNA expression levels of CB1/CB2 and Ly6G from the same sample by Pearson correlation test (Figure 2A). Although both CB1 and CB2 were positively correlated with Ly6G (*p* < 0.05), CB1 represented a particularly higher correlation coefficient (Figure 2A). Based on the prevailing amount of BM-derived neutrophils in injured liver, we used mouse neutrophils isolated from BM in subsequent cellular experiments. The expression of CB1 and CB2 in mouse BM derived-neutrophils were detected at mRNA level (Figure 2B), and protein level by immunofluorscence (Figure 2C). These results demonstrated the positive correlation between the expression of CB1 and neutrophil signatures in CCl_4_-treated mice and the abundant expression of CB1 in isolated neutrophils, suggesting that CB1 might play an important role in the recruitment and activation of neutrophils during sterile liver injury.

### 3.3. CB1 Rather than CB2 Mediates the Chemotaxis and Cytoskeletal Remodeling of Neutrophils In Vitro

**Figure 3:**
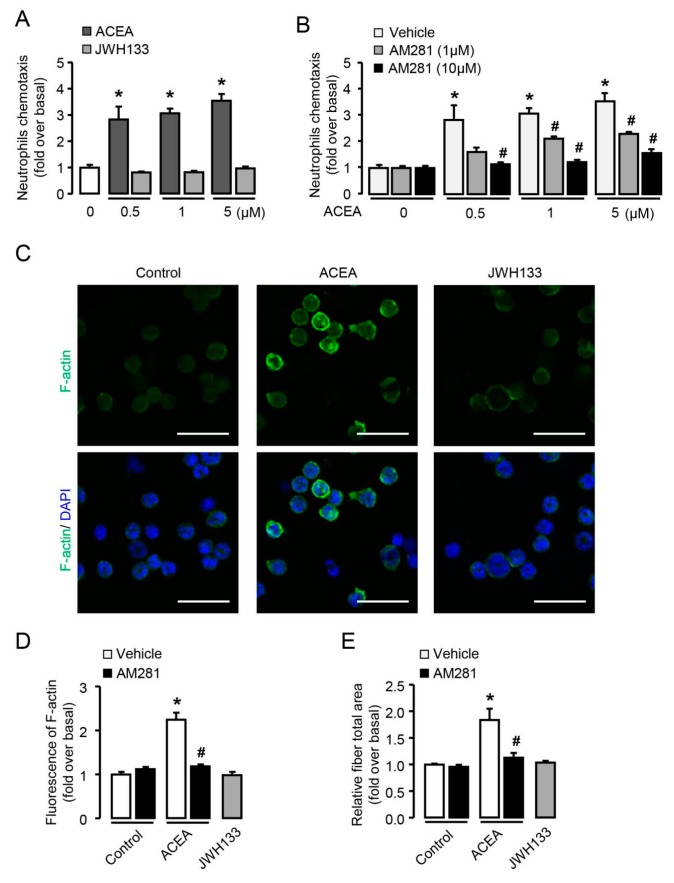
CB1 rather than CB2 mediates the chemotaxis and cytoskeletal remodeling of neutrophils in vitro. Chemotaxis assays were performed by transwell chambers. (**A**) Neutrophil chemotaxis with ACEA (CB1 agonist) or JWH133 (CB2 agonist) treatment for 2 h. (**B**) Effect of AM281 (CB1 antagonist) on neutrophil chemotaxis. (**C**) Representative images of F-actin remodeling with ACEA (1 μM, 2 h) and JWH 133 (1 μM, 2 h) treatment in neutrophils. Scale bars, 20 μm. (**D**) Quantification of F-actin with or without AM281 (10 μM) in ACEA-treated neutrophils. (**E**) The total fiber area was qualified by high content analysis in ACEA-treated neutrophils with or without AM281 pretreatment. Data are presented as the mean ± SEM. N = 5 per group. * *p* < 0.05 vs. control. # *p* < 0.05 vs. ACEA-treated alone.

Transwell assay was performed to explore whether CBs were involved in the chemotaxis of neutrophils in vitro. Treatment with ACEA (CB1 agonist) significantly increased the migration capacity of neutrophils in a dose-dependent manner, while JWH133 (CB2 agonist) had no such effect (Figure 3A). Due to the weak affinity between ACEA and CB2, we pretreated neutrophils with CB1 antagonist AM281 (1 and 10 μM) in ACEA-stimulated cells and showed declined chemotaxis of neutrophils with AM281 pretreatment (Figure 3B), further proving that CB1 mediated the chemotaxis of neutrophils.

Cytoskeletal remodeling is a prerequisite for cell chemotaxis and migration [29]. To evaluate the involvement of CB1 in cytoskeletal remodeling, neutrophils were stimulated with ACEA or JWH133 and then stained with FITC-conjugated phalloidin. Our findings indicated that ACEA-treated neutrophils were able to form a well-defined F-actin-rich leading edge (Figure 3C) and induced an increase in F-actin content (Figure 3D), whereas JWH133-treated neutrophils did not show obvious polymerized F-actin (Figure 3C,D). Consistent with the chemotaxis results above, AM281 could significantly reduce the increase of F-actin content induced by ACEA (Figure 3D). Moreover, the amount and distribution of actin fibers in neutrophils were determined by high content analysis. Treatment with ACEA showed significant increases in the total fiber area, which can be reversed by AM281 (Figure 3E). These results support that CB1 rather than CB2 plays an important role in neutrophil chemotaxis and cytoskeletal remodeling.

### 3.4. CB1 but not CB2 is Involved in the Activation of Neutrophils In Vitro

**Figure 4:**
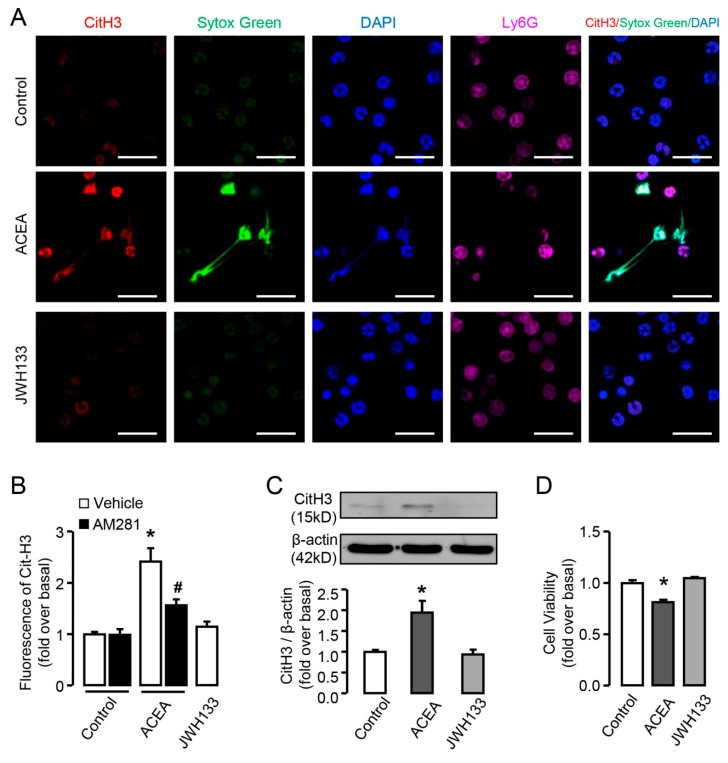
CB1 but not CB2 is involved in CitH3 expression and NETosis in vitro. (**A**) Representative confocal images of CitH3 (red) and Ly6G (violet) immunofluorescent staining and NETosis in ACEA or JWH133-treated neutrophils. The nuclei were stained with SYTOX^®^ Green (green) and DAPI (blue). Scale bars, 20 μm. (**B**) Quantification of CitH3 and NETosis in ACEA or JWH133-treated neutrophils. (**C**) CitH3 protein level in ACEA or JWH133-stimulated neutrophils was examined by Western blot. (**D**) Cell viability of neutrophils treated by ACEA or JWH-133 by CCK-8 assay. Data are presented as the mean ± SEM. N = 4 per group. * *p* < 0.05 vs. control. # *p* < 0.05 vs. ACEA-treated alone.

Next we sought to determine whether CBs played a role in the activation of neutrophils, including NETosis, MPO release from lysosome and ROS burst. Neutrophils stimulated with ACEA exhibited increased CitH3 fluorescence compared with untreated cells and represented manifest web-like chromatin release, in which chromatin and CitH3 (red) had good co-localization in neutrophils (Ly6G^+^, violet) (Figure 4A,B), and can be suppressed by AM281 (Figure 4B). Western blot results showed the same effect of ACEA on CitH3 expression (Figure 4C). In contrast, JWH133-treated neutrophils did not exhibit increased level of CitH3 or web-like chromatin release (Figure 4A–C). We performed CCK-8 assay to measure neutrophil viability under the treatment of ACEA, the condition to induce NETosis. Our results showed that ACEA decreased the cell viability of neutrophils, while CB2 agonist JWH133 had no such effect, which was in accordance with the results of NETosis (Figure 4D).

**Figure 5:**
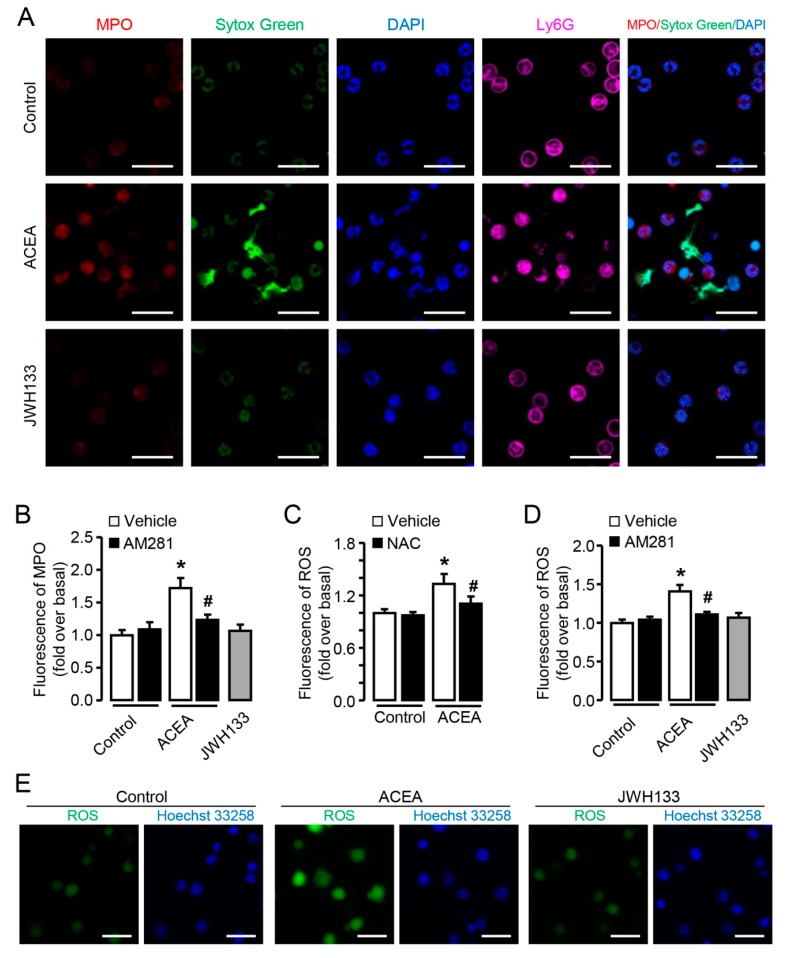
CB1 but not CB2 mediates MPO release and ROS burst in vitro. (**A**) Representative confocal images myeloperoxidase (MPO) (red) and Ly6G (violet) immunofluorescent staining in ACEA or JWH133-stimulated neutrophils. The nuclei were stained with SYTOX^®^ Green (green) and DAPI (blue). Scale bars, 20μm. (**B**) Quantification of MPO in ACEA or JWH133-treated neutrophils. (**C**) ROS burst in ACEA-treated neutrophils with or without NAC. (**D**) ROS burst in ACEA-treated neutrophils with or without AM281. (**E**) ROS immunofluorescence images in ACEA- or JWH-133-treated neutrophils. Data are presented as the mean ± SEM. N = 4 per group. * *p* < 0.05 vs. control. # *p* < 0.05 vs. ACEA-treated alone.

Normally, MPO exists in lysosome and is undetectable by antibodies, when stimulated MPO is released from lysosome becoming detectable [30]. Further we detected the release of MPO in neutrophils by immunofluorescence. ACEA treatment resulted in more MPO release of neutrophils compared with control, while JWH133 treatment had no such effect (Figure 5A,B). Similar to the results of CitH3, AM281 also blocked ACEA-induced MPO release in neutrophils (Figure 5B). We then measured ROS burst in neutrophils with ACEA treatment. In response to ACEA stimulation, neutrophils showed a significant increase of ROS burst at 10 min, and this increase could be significantly prevented by pre-incubation with NAC which is the scavenger of ROS (Figure 5C). In addition, pretreatment with AM281 repressed ACEA-induced ROS burst, and JWH133 could not induce ROS burst in neutrophils (Figure 5D). ROS immunofluorescence images also displayed the increase of ROS burst by ACEA treatment (Figure 5E). Altogether these results display that CB1 but not CB2 mediates the activation of neutrophils.

### 3.5. Blockade of CB1 Significantly Attenuates Neutrophil Infiltration and Liver Inflammation in CCl_4_-Treated Mice

**Figure 6:**
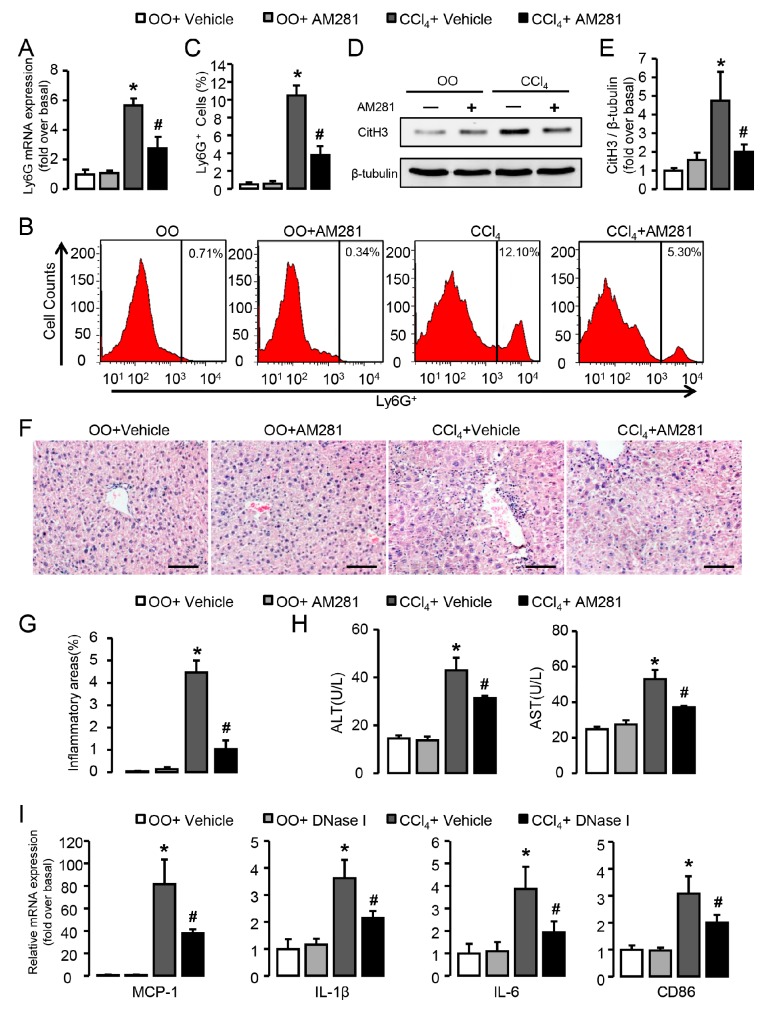
Blockade of CB1 significantly attenuates neutrophil infiltration and liver inflammation in CCl_4_-treated mice. (**A**) Ly6G mRNA expression in CCl_4_- or olive oil (OO)-treated liver with or without the administration of AM281. (**B**,**C**) Representative FACS plots and quantification for neutrophils in liver. (**D**,**E**) CitH3 protein levels in liver. (**F**) Representative H&E staining images of liver sections. Scale bars, 100 μm. (**G**) Quantitative analysis of liver inflammation areas. (**H**) Alanine aminotransferase (ALT) and aspartate aminotransferase (AST) levels were detected by chemistry analyzer. (**I**) mRNA expression of MCP-1, IL-1β, IL-6, and CD86 in CCl_4_- or OO-treated liver with or without the administration of DNase I. Data are presented as the mean ± SEM. N = 6 per group. * *p* < 0.05 vs. OO. # *p* < 0.05 vs. CCl_4_-treated alone.

The effects of CB1 blockade on neutrophil function and liver inflammation were further verified in vivo in mice treated with CCl_4_ for 2 weeks, when the hepatic levels of neutrophil signatures were highest. CB1 blockade by the administration of AM281 restrained the mRNA levels of Ly6G in injured livers (Figure 6A). In line with this, FACS analysis showed decreased neutrophils infiltration after AM281 administration in CCl_4_-treated mice compared with CCl_4_-treated alone (Figure 6B,C). Similarly, the down-regulated expression of CitH3 (Figure 6D,E) was observed in CCl_4_-treated mice with the administration of AM281, indicating less formation of NETs. Representative H&E-stained images showed a significant decrease of infiltrated inflammatory cells (Figure 6F) and the inflammatory area was quantified by digital image analysis (Figure 6G). Besides, AM281 administration protected liver against CCl_4_-induced injury with lower levels of alanine aminotransferase (ALT) and aspartate aminotransferase (AST) in mouse serum (Figure 6H). To elucidate the significance of NETs formation in liver inflamamtion, DNase I was administrated in CCl_4_-treated mice. Our results showed that DNase I significantly reduced mRNA expression of markers for liver inflammation (MCP1, IL-1β, IL-6) and macrophage activation (CD86) in the liver of CCl_4_-treated mice (Figure 6I). Altogether these results demonstrate that blockade of CB1 inhibits the recruitment and activation of neutrophils in the early stage of chronic liver injury and significantly attenuates liver injury. 

### 3.6. Gα_i/o_ Signal Is Involved in CB1-Mediated Chemotaxis and NETosis In Vitro

**Figure 7:**
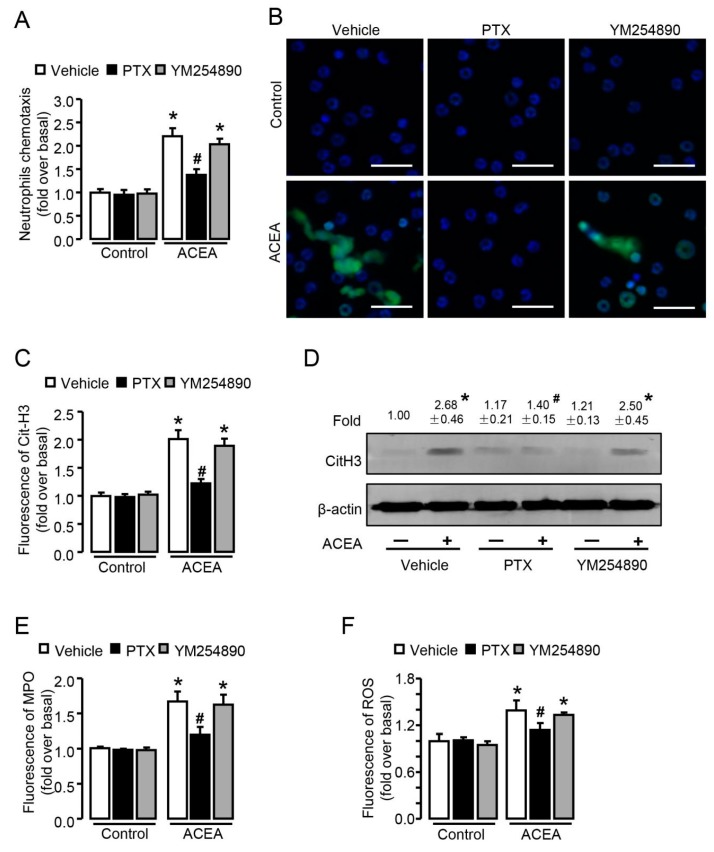
Gα_i/o_ signal is involved in CB1-mediated chemotaxis and NETosis in vitro. (**A**) ACEA-induced neutrophil chemotaxis pretreated with Gα_i/o_ inhibitor PTX or Gα_q_ inhibitor YM254890. (**B**,**C**) Representative images and quantification of CitH3 immunofluorescent staining (green) and NETosis in ACEA-treated neutrophils pretreated with PTX or YM254890. The nuclei were stained with DAPI (blue). Scale bars, 20 μm. (**D**) CitH3 protein level with PTX or YM254890 pretreatment was examined by Western blot. (**E**) Quantification of myeloperoxidase (MPO) immunofluorescence with pertussis toxin (PTX) or YM254890 pretreatment. (**F**) ROS burst in ACEA-treated neutrophils with or without PTX or YM254890 pretreatment. Data are presented as the mean ± SEM. N = 4 per group. * *p* < 0.05 vs. control. # *p* < 0.05 vs. ACEA-treated alone.

CB1 is a G-protein-coupled receptor which can transduce corresponding G protein-related signaling. To determine the distinct G-protein subtype involved in neutrophil chemotaxis and NETosis, we pre-treated cells with pertussis toxin (PTX) (Gα_i/o_ inhibitor) or YM254890 (Gα_q_ inhibitor). PTX prevented ACEA-induced neutrophil chemotaxis, while YM254890 exhibited no effect on it (Figure 7A). In line with this, the up-regulation of Cit-H3 protein induced by ACEA can be reversed by PTX, not YM254890 (Figure 7B–D). Moreover, less MPO release of neutrophils was observed after the pre-incubation of PTX in ACEA-stimulated cells compared with control, while YM254890 treatment had no such effect (Figure 7E). Markedly, PTX pretreatment inhibited ACEA-induced ROS burst, while YM254890 could not reduce ROS burst in neutrophils (Figure 7F). Taken together, these results indicate that Gα_i/o_, not Gα_q_ signal is involved in CB1-mediated chemotaxis and NETosis.

### 3.7. ROS Is Required for CB1-Mediated Neutrophil Chemotaxis and NETosis In Vitro

**Figure 8:**
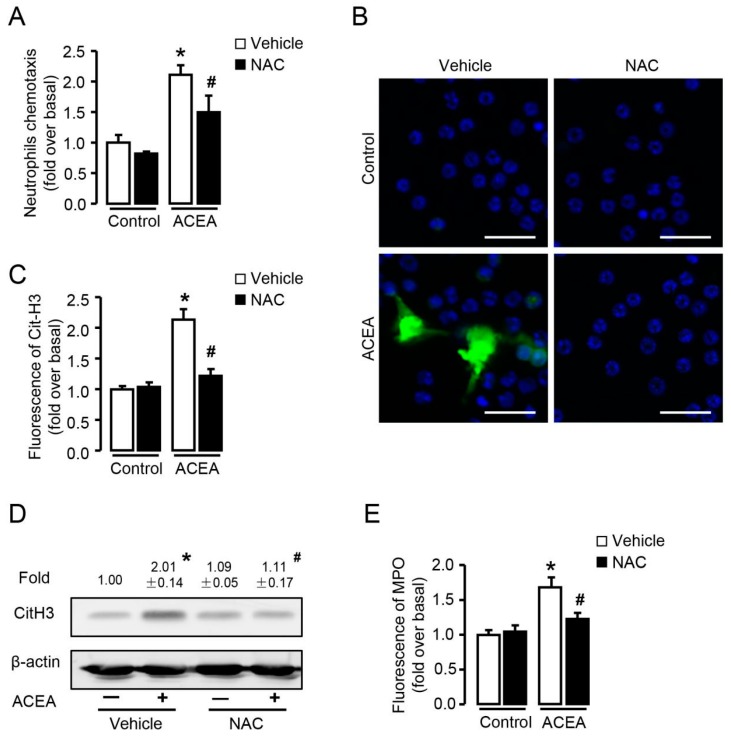
ROS is required in CB1-mediated chemotaxis and NETosis in vitro. (**A**) ACEA-induced neutrophil chemotaxis pretreated with or without NAC (5 mM). (**B**,**C**) Representative images and quantification of CitH3 immunofluorescent staining (green) and NETosis in ACEA-treated neutrophils pretreated with or without NAC. The nuclei were stained with DAPI (blue). Scale bars, 20 μm. (**D**) CitH3 protein level with or without NAC pretreatment was examined by Western blot. (**E**) Quantification of MPO immunofluorescence with or without NAC pretreatment. Data are presented as the mean ± SEM. N = 4 per group. * *p* < 0.05 vs. control. # *p* < 0.05 vs. ACEA-treated alone.

Since ROS burst was markedly induced by ACEA, we then explored whether ROS was involved in CB1-mediated neutrophil chemotaxis and NETosis. Transwell assay showed that CB1-mediated chemotaxis in neutrophils was suppressed by NAC (Figure 8A), suggesting that ROS acted as a signaling molecule in CB1-mediated neutrophil chemotaxis. As NETosis can either be ROS-dependent or ROS-independent [31,32], we pre-treated neutrophils with NAC before stimulation with ACEA and detected CitH3 expression and MPO release, to investigate whether CB1-induced NETosis required ROS in vitro. NAC significantly blocked ACEA-induced increase of CitH3 detected by immunofluorescence (Figure 8B,C) and Western blot (Figure 8D). Similarly, lower MPO fluorescence was observed in neutrophils pre-incubated with NAC before ACEA stimulation compared with that without NAC (Figure 8E). Collectively, elimination of ROS suppresses CB1-mediated chemotaxis and NETosis in neutrophils, suggesting that ROS acts as an important signaling molecule in CB1-mediated neutrophil function.

### 3.8. p38 MAPK Signaling Pathway, Located in the Downstream of ROS, Is Involved in CB1-Mediated Neutrophil Chemotaxis and NETosis

**Figure 9:**
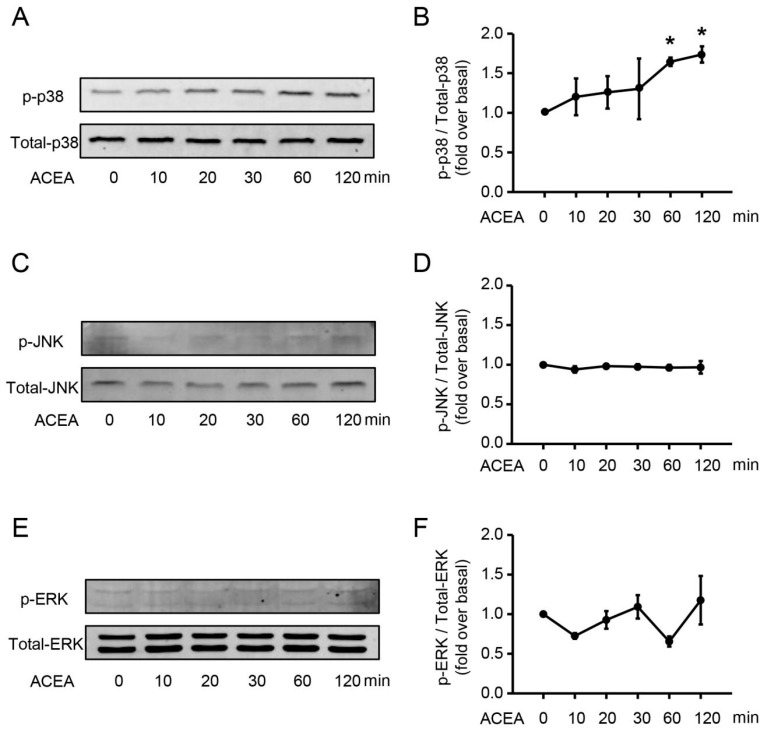
ACEA increases the protein level of phosphor-p38 in neutrophils. (**A**,**B**) Phosphor-p38 and total p38 expression after ACEA treatment was measured by Western blot. (**C**,**D**) Phosphor-JNK and total JNK expression. (**E**,**F**) Phosphor-ERK and total ERK expression.Data are presented as the mean ± SEM. N = 3 per group. * *p* < 0.05 vs. control.

CB1 is a G-protein-coupled receptor whose biological function depends on multiple signaling pathways, such as AMPK and MAPK signaling pathways [33]. To identify which pathway controls the chemotaxis and NETosis of neutrophils, we first detected the phosphorylation of p38, JNK and ERK after ACEA treatment. Stimulation with ACEA led to a significant increase in the protein level of phosphor-p38 (Figure 9A,B), but failed to activate JNK (Figure 9C,D) and ERK (Figure 9E,F) in neutrophils. Based on these, we focused on the key role of p38 in neutrophil function in the following experiments.

**Figure 10:**
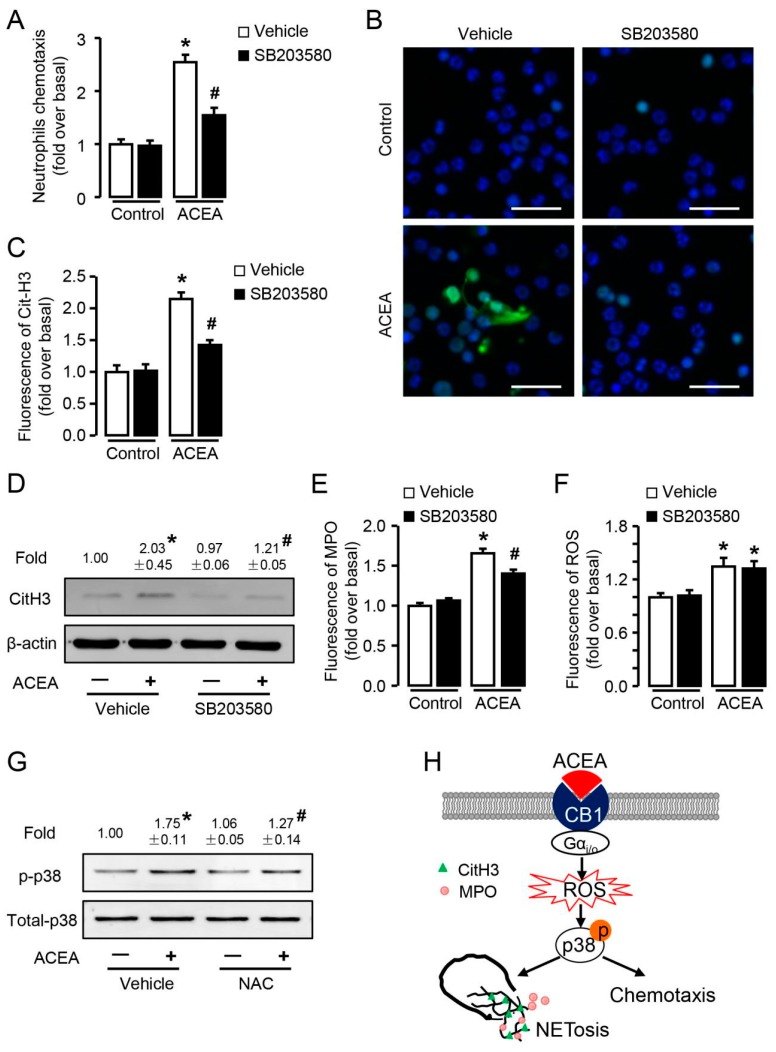
p38 MAPK signaling pathway is involved in CB1-mediated neutrophil chemotaxis and NETosis, and located in the downstream of ROS. (**A**) ACEA-induced neutrophil chemotaxis with or without p38 inhibitor SB203580 (10 μM). (**B**,**C**) Quantification of CitH3 immunofluorescence with or without SB203580. Scale bars, 20 μm. (**D**) CitH3 protein level with or without SB203580 was examined by Western blot. (**E**) Quantification of MPO immunofluorescence with or without SB203580. (**F**) ROS burst in ACEA-treated neutrophils with or without SB203580. (**G**) Phosphor-p38 and total p38 expression was measured by Western blot with or without NAC. (**H**) Schema graph of CB1 in neutrophil chemotaxis and NETosis. Data are presented as the mean ± SEM. N = 4 per group. * *p* < 0.05 vs. control. # *p* < 0.05 vs. ACEA-treated alone.

Moreover, ACEA-induced neutrophil chemotaxis was significantly suppressed by p38 inhibitor SB203580 (Figure 10A), indicating the key role of p38 in neutrophil chemotaxis. In case of CB1-mediated NETosis, p38 inhibition restrained CitH3 expression detected by both fluorescence (Figure 10B,C) and Western blot (Figure 10D). Similarly, lower MPO fluorescence was observed in neutrophils pre-incubated with p38 inhibitor before ACEA stimulation (Figure 10E). These results indicate that CB1-mediated p38 phosphorylation is involved in neutrophil chemotaxis and NETosis. Furthermore, inhibition of p38 had no effect on the production of ROS (Figure 10F), whereas elimination of ROS inhibited CB1-mediated phosphorylation of p38 (Figure 10G), suggesting that ROS was upstream of p38. Combining the above results, p38 MAPK signaling pathway is required for CB1-mediated neutrophil chemotaxis and NETosis, and locates in the downstream of ROS (Figure 10H).

## 4. Discussion

In summary, this study demonstrates for the first time that CB1 mediates neutrophil chemotaxis and activation in a ROS- and p38 MAPK-dependent manner in sterile liver inflammation. Our work provides several new findings as follows: 1. Numerous bone marrow-derived neutrophils are recruited and activated in the liver of CCl_4_-treated mice; 2. CBs positively correlate with neutrophil signatures in CCl_4_-treated mice, and are abundantly expressed in isolated neutrophils; 3. In vitro CB1 rather than CB2 mediates neutrophil chemotaxis, NETosis, MPO release and ROS burst via Gα_i/o_ signal; 4. ROS and p38 MAPK signaling pathway are both required for CB1-mediated neutrophil chemotaxis and NETosis, and p38 MAPK signaling pathway locates in the downstream of ROS; 5. Blockade of CB1 significantly attenuates neutrophil infiltration and liver inflammation in CCl_4_-treated mice. 

Neutrophils act as the first responders of the innate immune system and their crucial role in fighting invading pathogens during bacterial inflammation has been well established by published literature [34,35]. Recently more and more studies have been focusing on the prevailing role of neutrophils in sterile inflammation, and overexuberant neutrophil recruitment is associated with collateral tissue damage, defective healing, and chronic inflammation [36,37]. In the current study, we show that numerous BM-derived neutrophils are recruited to the site of liver injury shortly. The hepatic levels of neutrophil marker Ly6G begin to rise from 7 days of CCl_4_ administration and peek at 2 weeks, which is the early stage of chronic liver injury. This is in agreement with published studies demonstrating that neutrophil depletion by injection of Ly6G antibody markedly reduces chronic-binge ethanol feeding-induced liver injury and liver transplantation ischemia–reperfusion injury [8,38,39]. However, there are studies identifying the dual role for neutrophils in acetaminophen-induced acute liver injury, with neutrophil-mediated injury amplification early on, but exerting protective effects during the repair phase as depletion of neutrophils increases liver damage [40,41]. Also neutrophils contribute to spontaneous resolution of liver inflammation and fibrosis via microRNA-223 in CCl_4_-induced chronic liver injury [41]. More studies will be needed to figure out the mechanism underlying differential role of neutrophils in different models of liver inflammation and fibrosis.

ECS is implicated in the pathogenesis of numerous diseases, including cancer, cardiovascular disease, and liver disease [42,43]. Especially CB1 has emerged as a pivotal mediator in liver and exerts profibrogenic effects in chronic liver diseases including hepatic fibrosis, liver cirrhosis alcoholic fatty liver and nonalcoholic fatty liver [44]. Our previous studies have also demonstrated the vital role of CB1 in the migration and activation of BM-derived mesenchymal stromal cells and monocytes/macrophages in CCl_4_-induced chronic liver injury [26,27,45,46]. However, the effect of CBs on neutrophil function during sterile liver inflammation is unclear up to now and is first documented in the present study. Our data display that CB1 rather than CB2 mediates the chemotaxis of neutrophils and NETosis, and CB1 blockade with AM281 reduces the infiltration and NETosis of neutrophils and attenuates liver injury in vivo, which can be used as a novel target for the treatment of liver fibrosis.

NETs, DNA webs released into the extracellular environment by activated neutrophils, are thought to play a key role in the function of neutrophils [47,48]. Unlike nuclear chromatin, NETs are highly decondensed chromatin structures, and PAD4 has been reported to be essential in chromatin decondensation to form NETs by catalyzing histone citrullination [49]. The partial PAD4-deficiency (Pad4^+/–^) reduced acute lung injury induced by bacteria and improved survival, while complete NET inhibition by PAD4 deficiency (Pad4^–/–^) reduced lung injury [50]. Further studies will be needed to investigate PAD4 expression and the effect of PAD4 on NETosis in our CCl_4_-treated mice and ACEA-treated neutrophils.

NETosis was initially found dependent on the ROS by NADPH [32], and was subsequently found also to be independent of ROS [31]. In the present study, ACEA-mediated neutrophil chemotaxis and NETosis can be significantly suppressed by ROS scavenger NAC, indicating that CB1 induces the chemotaxis and NETosis of neutrophils in a ROS-dependent manner. Since ROS could activate MAPK pathway and then mediate PMA-induced NETosis [51], here we detect the activation of p38, JNK, and ERK after ACEA stimulation, showing that only p38 MAPK pathway is activated and involved in CB1-mediated neutrophil chemotaxis and NETosis. Further we identify the upstream and downstream relationship of ROS and p38 MAPK signaling pathways by the fact that p38 inhibition has no effect on the production of ROS, whereas ROS elimination inhibits CB1-mediated phosphorylation of p38, suggesting that ROS acts as an upstream signaling molecule of p38 MAPK in neutrophil chemotaxis and NETosis. 

In conclusion, we identify the critical role of CB1 in neutrophil chemotaxis and NETosis during sterile liver inflammation and explore the underlying mechanism associated with Gα_i/o_/ROS/p38 MAPK signaling pathway, which may open new perspectives for pharmacological treatment of liver disease.

## References

[1] Bardoel B.W., Kenny E.F., Sollberger G., Zychlinsky A. (2014). The balancing act of neutrophils. Cell Host Microbe.

[2] Jorch S.K., Kubes P. (2017). An emerging role for neutrophil extracellular traps in noninfectious disease. Nat. Med..

[3] Cools-Lartigue J., Spicer J., Najmeh S., Ferri L. (2014). Neutrophil extracellular traps in cancer progression. Cell. Mol. Life Sci..

[4] Metzler K.D., Goosmann C., Lubojemska A., Zychlinsky A., Papayannopoulos V. (2014). A myeloperoxidase-containing complex regulates neutrophil elastase release and actin dynamics during NETosis. Cell Rep..

[5] Sun L., Wu Q., Nie Y., Cheng N., Wang R., Wang G., Zhang D., He H., Ye R.D., Qian F. (2018). A Role for MK2 in Enhancing Neutrophil-Derived ROS Production and Aggravating Liver Ischemia/Reperfusion Injury. Front. Immunol..

[6] de Bont C.M., Koopman W., Boelens W.C., Pruijn G. (2018). Stimulus-dependent chromatin dynamics, citrullination, calcium signalling and ROS production during NET formation. Biochim. Biophys. Acta Mol. Cell Res..

[7] Brinkmann V., Reichard U., Goosmann C., Fauler B., Uhlemann Y., Weiss D.S., Weinrauch Y., Zychlinsky A. (2004). Neutrophil extracellular traps kill bacteria. Science.

[8] Huang H., Tohme S., Al-Khafaji A.B., Tai S., Loughran P., Chen L., Wang S., Kim J., Billiar T., Wang Y. (2015). Damage-associated molecular pattern-activated neutrophil extracellular trap exacerbates sterile inflammatory liver injury. Hepatology.

[9] Sollberger G., Tilley D.O., Zychlinsky A. (2018). Neutrophil Extracellular Traps: The Biology of Chromatin Externalization. Dev. Cell.

[10] Hisada Y., Grover S.P., Maqsood A., Houston R., Ay C., Noubouossie D.F., Cooley B.C., Wallen H., Key N.S., Thalin C. (2019). Neutrophils and neutrophil extracellular traps enhance venous thrombosis in mice bearing human pancreatic tumors. Haematologica.

[11] Castanheira F., Kubes P. (2019). Neutrophils and NETs in modulating acute and chronic inflammation. Blood.

[12] Grabcanovic-Musija F., Obermayer A., Stoiber W., Krautgartner W.D., Steinbacher P., Winterberg N., Bathke A.C., Klappacher M., Studnicka M. (2015). Neutrophil extracellular trap (NET) formation characterises stable and exacerbated COPD and correlates with airflow limitation. Respir. Res..

[13] Knight J.S., Subramanian V., O’Dell A.A., Yalavarthi S., Zhao W., Smith C.K., Hodgin J.B., Thompson P.R., Kaplan M.J. (2015). Peptidylarginine deiminase inhibition disrupts NET formation and protects against kidney, skin and vascular disease in lupus-prone MRL/lpr mice. Ann. Rheum. Dis..

[14] Mistry P., Carmona-Rivera C., Ombrello A.K., Hoffmann P., Seto N.L., Jones A., Stone D.L., Naz F., Carlucci P., Dell’Orso S. (2018). Dysregulated neutrophil responses and neutrophil extracellular trap formation and degradation in PAPA syndrome. Ann. Rheum. Dis..

[15] Yazdani H.O., Chen H.W., Tohme S., Tai S., van der Windt D.J., Loughran P., Rosborough B.R., Sud V., Beer-Stolz D., Turnquist H.R. (2017). IL-33 exacerbates liver sterile inflammation by amplifying neutrophil extracellular trap formation. J. Hepatol..

[16] He Y., Feng D., Li M., Gao Y., Ramirez T., Cao H., Kim S.J., Yang Y., Cai Y., Ju C. (2017). Hepatic mitochondrial DNA/Toll-like receptor 9/MicroRNA-223 forms a negative feedback loop to limit neutrophil overactivation and acetaminophen hepatotoxicity in mice. Hepatology.

[17] Mallat A., Lotersztajn S. (2016). Targeting cannabinoid receptors in hepatocellular carcinoma?. Gut.

[18] Fonseca B.M., Costa M.A., Almada M., Correia-da-Silva G., Teixeira N.A. (2013). Endogenous cannabinoids revisited: A biochemistry perspective. Prostag. Other Lipid Med..

[19] Rossi F., Tortora C., Punzo F., Bellini G., Argenziano M., Di Paola A., Torella M., Perrotta S. (2019). The Endocannabinoid/Endovanilloid System in Bone: From Osteoporosis to Osteosarcoma. Int. J. Mol. Sci..

[20] Rossi F., Punzo F., Umano G.R., Argenziano M., Miraglia D.G.E. (2018). Role of Cannabinoids in Obesity. Int. J. Mol. Sci..

[21] Starowicz K., Przewlocka B. (2012). Modulation of neuropathic-pain-related behaviour by the spinal endocannabinoid/endovanilloid system. Philos. Trans. R. Soc. Lond. B Biol. Sci..

[22] Mallat A., Teixeira-Clerc F., Lotersztajn S. (2013). Cannabinoid signaling and liver therapeutics. J. Hepatol..

[23] Tam J., Vemuri V.K., Liu J., Batkai S., Mukhopadhyay B., Godlewski G., Osei-Hyiaman D., Ohnuma S., Ambudkar S.V., Pickel J. (2010). Peripheral CB1 cannabinoid receptor blockade improves cardiometabolic risk in mouse models of obesity. J. Clin. Investig..

[24] Gary-Bobo M., Elachouri G., Gallas J.F., Janiak P., Marini P., Ravinet-Trillou C., Chabbert M., Cruccioli N., Pfersdorff C., Roque C. (2007). Rimonabant reduces obesity-associated hepatic steatosis and features of metabolic syndrome in obese Zucker fa/fa rats. Hepatology.

[25] Chen M., Hou P., Zhou M., Ren Q., Wang X., Huang L., Hui S., Yi L., Mi M. (2019). Resveratrol attenuates high-fat diet-induced non-alcoholic steatohepatitis by maintaining gut barrier integrity and inhibiting gut inflammation through regulation of the endocannabinoid system. Clin. Nutr..

[26] Tian L., Li W., Yang L., Chang N., Fan X., Ji X., Xie J., Yang L., Li L. (2017). Cannabinoid Receptor 1 Participates in Liver Inflammation by Promoting M1 Macrophage Polarization via RhoA/NF-kappaB p65 and ERK1/2 Pathways, Respectively, in Mouse Liver Fibrogenesis. Front. Immunol..

[27] Mai P., Yang L., Tian L., Wang L., Jia S., Zhang Y., Liu X., Yang L., Li L. (2015). Endocannabinoid System Contributes to Liver Injury and Inflammation by Activation of Bone Marrow-Derived Monocytes/Macrophages in a CB1-Dependent Manner. J. Immunol..

[28] Han Z., Zhu T., Liu X., Li C., Yue S., Liu X., Yang L., Yang L., Li L. (2012). 15-deoxy-Delta12,14 -prostaglandin J2 reduces recruitment of bone marrow-derived monocyte/macrophages in chronic liver injury in mice. Hepatology.

[29] Malinova T.S., Huveneers S. (2018). Sensing of Cytoskeletal Forces by Asymmetric Adherens Junctions. Trends Cell Biol..

[30] Papayannopoulos V., Metzler K.D., Hakkim A., Zychlinsky A. (2010). Neutrophil elastase and myeloperoxidase regulate the formation of neutrophil extracellular traps. J. Cell Biol..

[31] Pilsczek F.H., Salina D., Poon K.K., Fahey C., Yipp B.G., Sibley C.D., Robbins S.M., Green F.H., Surette M.G., Sugai M. (2010). A novel mechanism of rapid nuclear neutrophil extracellular trap formation in response to Staphylococcus aureus. J. Immunol..

[32] Fuchs T.A., Abed U., Goosmann C., Hurwitz R., Schulze I., Wahn V., Weinrauch Y., Brinkmann V., Zychlinsky A. (2007). Novel cell death program leads to neutrophil extracellular traps. J. Cell Biol..

[33] Turu G., Hunyady L. (2010). Signal transduction of the CB1 cannabinoid receptor. J. Mol. Endocrinol..

[34] Kolaczkowska E., Kubes P. (2013). Neutrophil recruitment and function in health and inflammation. Nat. Rev. Immunol..

[35] Meyer E., Beyersmann J., Bertz H., Wenzler-Rottele S., Babikir R., Schumacher M., Daschner F.D., Ruden H., Dettenkofer M. (2007). Risk factor analysis of blood stream infection and pneumonia in neutropenic patients after peripheral blood stem-cell transplantation. Bone Marrow Transpl..

[36] Peiseler M., Kubes P. (2019). More friend than foe: The emerging role of neutrophils in tissue repair. J. Clin. Investig..

[37] Phillipson M., Kubes P. (2011). The neutrophil in vascular inflammation. Nat. Med..

[38] Bertola A., Park O., Gao B. (2013). Chronic plus binge ethanol feeding synergistically induces neutrophil infiltration and liver injury in mice: A critical role for E-selectin. Hepatology.

[39] Klune J.R., Bartels C., Luo J., Yokota S., Du Q., Geller D.A. (2018). IL-23 mediates murine liver transplantation ischemia-reperfusion injury via IFN-gamma/IRF-1 pathway. Am. J. Physiol. Gastrointest. Liver Physiol..

[40] Alvarenga D.M., Mattos M.S., Lopes M.E., Marchesi S.C., Araujo A.M., Nakagaki B.N., Santos M.M., David B.A., De Souza V.A., Carvalho E. (2018). Paradoxical Role of Matrix Metalloproteinases in Liver Injury and Regeneration after Sterile Acute Hepatic Failure. Cells-Basel.

[41] Calvente C.J., Tameda M., Johnson C.D., Del P.H., Lin Y.C., Adronikou N., De Mollerat D.J.X., Llorente C., Boyer J., Feldstein A.E. (2019). Neutrophils contribute to spontaneous resolution of liver inflammation and fibrosis via microRNA-223. J. Clin. Investig..

[42] Basu P.P., Aloysius M.M., Shah N.J., Brown R.J. (2014). Review article: The endocannabinoid system in liver disease, a potential therapeutic target. Aliment. Pharmacol. Ther..

[43] Miller L.K., Devi L.A. (2011). The highs and lows of cannabinoid receptor expression in disease: Mechanisms and their therapeutic implications. Pharmacol. Rev..

[44] Mahmoud M.F., Swefy S.E., Hasan R.A., Ibrahim A. (2014). Role of cannabinoid receptors in hepatic fibrosis and apoptosis associated with bile duct ligation in rats. Eur. J. Pharmacol..

[45] Wang L., Yang L., Tian L., Mai P., Jia S., Yang L., Li L. (2017). Cannabinoid Receptor 1 Mediates Homing of Bone Marrow-Derived Mesenchymal Stem Cells Triggered by Chronic Liver Injury. J. Cell. Physiol..

[46] Mai P., Tian L., Yang L., Wang L., Yang L., Li L. (2015). Cannabinoid receptor 1 but not 2 mediates macrophage phagocytosis by G(alpha)i/o /RhoA/ROCK signaling pathway. J. Cell. Physiol..

[47] Liew P.X., Kubes P. (2019). The Neutrophil’s Role During Health and Disease. Physiol. Rev..

[48] Pfeiler S., Stark K., Massberg S., Engelmann B. (2017). Propagation of thrombosis by neutrophils and extracellular nucleosome networks. Haematologica.

[49] Li P., Li M., Lindberg M.R., Kennett M.J., Xiong N., Wang Y. (2010). PAD4 is essential for antibacterial innate immunity mediated by neutrophil extracellular traps. J. Exp. Med..

[50] Lefrancais E., Mallavia B., Zhuo H., Calfee C.S., Looney M.R. (2018). Maladaptive role of neutrophil extracellular traps in pathogen-induced lung injury. JCI Insight.

[51] Keshari R.S., Verma A., Barthwal M.K., Dikshit M. (2013). Reactive oxygen species-induced activation of ERK and p38 MAPK mediates PMA-induced NETs release from human neutrophils. J. Cell. Biochem..

